# Free Radical Scavenging Activity and Comparative Metabolic Profiling of *In Vitro* Cultured and Field Grown *Withania somnifera* Roots

**DOI:** 10.1371/journal.pone.0123360

**Published:** 2015-04-14

**Authors:** Kalaiselvi Senthil, Pankajavalli Thirugnanasambantham, Taek Joo Oh, So Hyun Kim, Hyung Kyoon Choi

**Affiliations:** 1 Department of Biochemistry, Biotechnology and Bioinformatics, Avinashilingam University for Women, Coimbatore, 641043, India; 2 College of Pharmacy, Chung-Ang University, Seoul, 156–756, Republic of Korea; National Research Council of Italy, ITALY

## Abstract

Free radical scavenging activity (FRSA), total phenolic content (TPC), and total flavonoid content (TFC) of *in vitro* cultured and field grown *Withania somnifera* (Ashwagandha) roots were investigated. Withanolides analysis and comprehensive metabolic profiling between 100% methanol extracts of *in vitro* and field grown root tissues was performed using high performance thin layer chromatography (HPTLC) and gas chromatography-mass spectrometry (GC-MS), respectively. Significantly higher levels of FRSA, TPC, and TFC were observed in *in-vitro* cultured roots compared with field grown samples. In addition, 30 day-cultured *in vitro* root samples (1MIR) exhibited a significantly higher FRSA (IC_50_ 81.01 μg/mL), TPC (118.91 mg GAE/g), and TFC (32.68 mg CE/g) compared with those in 45 day-cultured samples (1.5MIR). Total of 29 metabolites were identified in *in vitro* cultured and field grown roots by GC-MS analysis. The metabolites included alcohols, organic acids, purine, pyrimidine, sugars, and putrescine. Vanillic acid was only observed in the *in vitro* cultured root samples, and higher level of the vanillic acid was observed in 1MIR when compared to 1.5MIR. Therefore, it is suggested that 1MIR might serve as an alternative to field grown roots for the development of medicinal and functional food products.

## Introduction

*Withania somnifera*, commonly known as Ashwagandha, is a traditional medicinal herb that has been used since ancient times. Various forms of the herb including decoctions, infusions, ointments, powders, and syrups are used as medicine all over the world for patients of all age groups without any side effects, even during pregnancy [1–5]. Various bioactive constituents of this plant have been reported to possess adaptogenic, anticancer, anticonvulsant, immunomodulatory, antioxidative, and neurological effects [6].

The major biochemical constituents of this plant include steroidal alkaloids and steroidal lactones, which constitute a class of compounds known as withanolides, naturally occurring C28- steroidal lactones built on an intact or rearranged ergostane framework, in which C-22 and C-26 are appropriately oxidized to form a six-membered lactone ring [7,8]. Among withanolides, withanolide A and withaferin A were reported to be dominant metabolites, distributed among numerous plant tissues at varying concentrations, and were shown to possess many therapeutic properties [9–11]. Despite the therapeutic advantages that continuously attract the attention of pharmacologists, the annual production of this plant is not sufficient to meet the global demand [12]. Therefore, an *in vitro* culture system could serve as an alternative to field grown plants for the production of medically valuable compounds. *In vitro* cultures have a tendency to produce secondary metabolites faster than field-grown plants, owing to their active growth [13].

In the last decade the metabolomic analysis of medicinal plants has been well established [14–16]. Metabolomics in this field has been used for quality control [17–19], identification of compounds [20,21], and correlation of a plant’s metabolome with biological activity [22,23]. The improvement in the analytical performance greatly facilitates the development of plant metabolomics. Metabolomics platforms including nuclear magnetic resonance (NMR) spectrometry, liquid chromatography mass spectrometry (LC-MS), and gas chromatography mass spectrometry (GC-MS) detect a wide range of metabolites resulting in comprehensive data sets. Among these techniques, GC-MS facilitates the identification and robust quantification of various metabolites from plant extract. Furthermore, GC-MS has long been used for metabolomics approach and thus the protocols and the libraries were well established [24]. Although LC-MS has a relatively broad coverage of metabolites compared with GC-MS, it has a distinct drawbacks, such as lower reproducibility of retention times and more susceptible to ion suppression effects, which hamper quantification [15].

There have been no reports regarding the comprehensive metabolic profiling and free radical scavenging activity as well as total phenolics contents (TPC) and total flavonoids content (TFC) of field-grown and *in vitro* cultured *W*. *somnifera* roots. Thus, the purpose of this study was to investigate the metabolic profiles and free radical scavenging activities of field and *in vitro* cultured *W*. *somnifera* roots.

## Materials and Methods

### Solvents and Chemicals

HPLC-grade methanol, water, Murashige and Skoog (MS) powder, pyridine, 1,1-diphenyl-2-picrylhydrazyl (DPPH), ascorbic acid, dimethyl sulfoxide (DMSO), Folin-Ciocalteu’s reagent, sodium carbonate, gallic acid, sodium nitrite, aluminum chloride, Catechin, and methoxyamine were purchased from Sigma (St. Louis, MO, USA). BSTFA [*N*,*O*-bis(trimethylsilyl)trifluoroacetamide containing 1% trimethyl chlorosilane (TMCS)] was obtained from Alfa Aesar (Ward Hill, MA, USA), and 2-Chloronaphtahalene (internal standard for GC-MS) was purchased from Tokyo Chemical Industry Co., Ltd. (Tokyo, Japan).

### Plant Materials

Seeds of *W*. *somnifera* (L.) Dunal “Jawahar” were obtained from the Central Institute of Medicinal and Aromatic Plants (Lucknow). They were surface sterilized as per the procedure described by Murthy et al.[25] and were inoculated in Murashige and Skoog (MS) solid basal medium supplemented with 2% sucrose for germination, and were subsequently incubated in the dark at 25°C. Shoots from *in vitro* germinated seedlings were cultured in basal MS media and were maintained at 25°C for a 16 h photoperiod. These plants were maintained in MS basal medium for at least four generations.

For root induction, fully grown leaf explants grown *in vitro* on MS medium were excised and trimmed into pieces of about 1 cm^2^ and were inoculated on a MS solid medium (0.8% agar) supplemented with 3% sucrose and 2 mg L^-1^ IBA+0.5 mg L^-1^ IAA [26]. The pH of the medium was adjusted to 5.6 ± 0.2 prior to sterilization. The inoculated explants were incubated at 25°C under a 16 h photoperiod for root induction.

For mass production, 500 mg of the root tips and branches from *in vitro*-induced adventitious roots were inoculated into one liter of liquid MS media containing 3% sucrose, without any plant hormone in a 2 L bubble column bioreactor (Biopia, Republic of Korea), which aseptically aerated with 0.1 vvm aeration rate using a mini aerator (Biopia, Republic of Korea) through 0.20 μm hydrophobic PTFE (Sartorius stedim) membrane filter. The temperature was maintained at 25°C and the media was changed once every 15 days. Three bioreactors were used to obtain triplicate biological replications. After 30 days (1MIR: 1 month *in vitro* root, *in vitro* cultured root for 30 days) and 45 days (1.5MIR: 1.5 month *in vitro* root, *in vitro* cultured root for 45 days) of inoculation, *in vitro* root tissues were harvested for further analysis.

To obtain field grown root samples, the same seeds were sown in the field following standard cultivation practices. The seeds were sown in the month of August, which has been known as ideal plantation time, in the field of Avinashilingam Institute, Coimbatore, Tamilnadu. The field lies between 11°1’ North latitude and 76°57’ East longitude. The city receives an annual rainfall of around 700 mm from September to October with the north east and south west monsoons. The seeds are sown about 2 cm deep into the soil at a distance of about 50 cm. The method of sowing was preferred as it promotes the development of a health root system compared to being sown using the broad casting method. The temperature range was 28–30°C. There was no application of fungicides or organic fertilizers. The field was irrigated twice in a week. The root samples grown in the field were harvested after 60 days (2MFR: 2 month field-grown root, field grown root for 60 days) and 150 days (5MFR: 5 month field-grown root, field grown root for 150 days) for further analysis.

### Withanolide A and Withaferin A Analysis

One gram of freeze-dried and powdered root material was extracted with 50 ml of 100% methanol. The extraction was carried out four times. Each time the extract was sonicated for 20 min, then kept in shaker for 2 hr at 100 rpm and filtered using Whatmann No. 1 filter paper. All extracts were then pooled, filtered and evaporated to dryness using a rotary vacuum evaporator (Roteva, Mumbai) in a water bath at 40°C. The residue was dissolved in 10 ml of HPLC grade methanol and stored at -20°C until further analysis. Withanolide A and withaferin A standards were obtained from Chromodex (city, USA). Standard stock solutions of withanolide A and withaferin A (1.0 mg/ml) were prepared using HPLC grade methanol and stored in a refrigerator at 4°C. From the stock solutions, working solutions (0.1 mg/ml) were prepared by dilution with HPLC grade methanol. High performance thin layer chromatography (HPTLC) analysis was carried out using toluene:ethyl acetate:formic acid in the ratio 5:5:1 (v/v/v). Chromatography was performed at 25±2°C on precoated aluminium plates (20x10 cm/10x10cm and 0.2 mm thickness). The withanolide-A and withaferin-A standards at concentration of 0.1 mg/ml were applied with the concentration range from 200 to 1000 ng per band for quantification. A volume of 20 μl of samples dissolved in HPLC grade methanol along with the standards were applied to the plates as 6/8 mm bands, 8 mm from the bottom, 15 mm from the side, under a stream of nitrogen, by means of a CAMAG Linomat V semiautomatic sample applicator (Camag Chemie, Muttenz, Switzerland) fixed with a 100 μl HPTLC syringe (Hamilton Co., NV, USA). The spraying rate was 150 nLs^-1^. Linear ascending development to a distance of 80 mm was carried out on 10x10 cm/20x20 cm twin trough chamber saturated with the mobile phase, pre-saturated with the solvent for 30 min. After running, the plates were removed from the chamber, air dried and visualized at 254 and 366 nm. Densitometric scanning was performed with Camag TLC scanner III (Camag Chemie, Muttenz, Switzerland) controlled by CAMAG CATS 4 integration software at 235 nm for withanolide A and withaferin A. The slit dimensions were 4x0.3/6x0.3 mm and the scanning speed was 20 mm s^-1^. The plates were derivatized in anisaldehyde-sulphuric acid reagent (conc. sulfuric acid: methanol: glacial acetic acid: anisaldehyde in the ratio of 5:85:10:0.5) for 2 seconds and kept in hot-air oven for 10 min at 110°C for detection of spots. The R_f_ values of the resolved spots were noted. The amount of withaferin A and withanolide A was computed from peak areas in all samples.

### Free Radical Scavenging Activity

The 1,1-diphenyl-2-picrylhydrazyl (DPPH) radical scavenging activity was determined using a previously reported method with minor modifications [27]. The samples extracted with 100% methanol and ascorbic acid (positive control) were dissolved in dimethyl sulfoxide (DMSO). An aliquot (20 μL) of each of the extracted samples was introduced into a 96 well plate, and 180 μL of 100 mM DPPH solution was added. After standing for 30 min at room temperature, the absorbance was measured at 517 nm using microplate spectrophotometer (xMark, Biorad, Berkeley, CA). DMSO was utilized as the blank. The free radical scavenging activity was expressed as IC_50_ (the concentration of the *Withania somnifera* roots sample (μg/mL) required to scavenge 50% of DPPH), and calculated from the calibration curve prepared by free radical scavenging activity percentage of the various concentrations of the extracts (10–500 μg/mL). The IC_50_ of the reference antioxidant ascorbic acid (1–20 μg/mL) was calculated by the same method as described above.

### Determination of Total Phenolic Content

The total phenolic content (TPC) was estimated by the modified Folin-Ciocalteu method [28]. All extracted samples were dissolved in DMSO. A 0.5 mL aliquot of each sample (1 mg/mL concentration) was transferred into a test tube, and 4.5 mL of distilled water and 0.5 mL of Folin-Ciocalteu’s reagent were added. After standing for 2 min at room temperature, 1.5 mL of 20% (v/w) sodium carbonate solution was added. The mixture was allowed to react in the dark for 1.5 h, and the absorbance at 765 nm was subsequently measured using microplate spectrophotometer (xMark, Biorad, Berkeley, CA). A calibration curve for gallic acid was prepared in the concentration range of 100–500 μg/mL. The results were expressed as milligrams of gallic acid equivalent per gram of the dried extract.

### Determination of Total Flavonoid Content

The total flavonoid content (TFC) was determined as per the method described previously [29]. All extracted samples were dissolved in DMSO. Briefly, 0.5 mL of each sample (1 mg/mL concentration) was mixed with 0.3 mL of 5% sodium nitrite. After standing for 5 min at room temperature, 0.3 mL of 10% aluminum chloride was added, and the sample was incubated for 6 min. Finally, 2 mL of 1M sodium hydroxide was added and the total volume of the mixture was adjusted to 5 mL by adding 1.9 mL of deionized water. Catechin was used as the standard for the calibration curve (10–100 μg/mL). The results were expressed as milligrams of catechin equivalent per gram of the dried extract.

### GC-MS Analysis

Dried samples (20 mg) of young and mature roots of *in vitro* cultured and field grown *W*. *somnifera* were extracted with 1 mL of 100% methanol prior to metabolite analysis by GC-MS. The extracts were sonicated for 30 min, followed by centrifugation at 2,000 rpm for 5 min. The supernatant was filtered through a 0.45 μm filter (PTFE, Sartorius Stedim Biotech, Göttingen, Germany). To perform derivatization of the extracted sample, 100 μL of each sample was transferred into a GC vial and dried with a flow of nitrogen gas. 30 μL of methoxylamine hydrochloride (200 μg/mL) in pyridine, 50 μL of BSTFA (*N*,*O*-bis(trimethylsilyl)trifluoroacetamide; Alfa Aesar, Ward Hill, MA, USA) containing 1% TMCS (trimethyl chlorosilane), and 10 μL of 2-chloronaphthalene (Tokyo Chemical Industry Co., Ltd., Tokyo, Japan; 200 μg/mL in pyridine as an internal standard) were added to dried vials. After derivatization, the samples were incubated for 60 min at 60°C, and were subsequently subjected to GC-MS analysis.

GC-MS analysis was performed using a 7890A Agilent GC system (Agilent Technologies, CA, USA) equipped with a 5975C mass selective detector (Agilent Technologies) and automatic sampler (7683 B series, Agilent Technologies). Electron impact ionization mode with an ionization energy of 70 eV was used for GC-MS detection. Analytes were separated on a fused silica capillary column with 5% phenyl methylpolysiloxane (DB-5, Agilent Technologies; dimensions were 30 m × 0.25 mm i.d. × 0.25 μm film thickness). Helium was used as the carrier gas at a constant flow rate of 1.0 mL/min. The inlet temperature was set to 250°C, with an injection volume of 1.0 μL and split ratio of 1:10. The mass range was 50–700 Da and data were obtained in mass selective detector with the full scan mode. The oven temperature was set to 70°C and was programmed to increase to 150°C (at 5°C/min) then 250°C (at 3°C/min; hold 2 min), and finally 320°C (at 10°C/min; hold 3 min).

### Data Processing

Pearson correlation test was conducted using IBM SPSS Statistics 19 software (IBM, Somers, NY). To quantitatively compare the global metabolic profile of all samples, raw datasets from GC-MS analysis were processed as described previously [30]. Mass spectra were deconvoluted using the AMDIS (Automated Mass Spectral Deconvolution and Identification System, http://chemdata.nist.gov/mass-spc/amdis/) and ELU files were created and analyzed with an online peak-filtering algorithm (SpectConnect, http://spectconnect.mit.edu). The metabolites were identified by comparing the mass spectra with those of the NIST-Wiley Mass Spectra Library. Then the normalization was performed by dividing the peak intensity of each compound by that of the internal standard for relative quantification of the metabolites in each sample. Significant differences in metabolite levels were detected by one-way analysis of variance (ANOVA) using Statistical Package for the Social Sciences (SPSS) software (version 19, IBM, Somers, NY) followed by Tukey’s significant-difference test. The level of statistical significance was set at *p*<0.05. Principal component analysis (PCA) was performed using SIMCA-P^+^ software (version 13.0, Umetrics, Umeå, Sweden). All data were mean-centered, with scaling to unit variance as a preprocessing method.

## Results and Discussion

### 1. Withaferin and Withanolide Content

In this study, two major withanolide contents in *in vitro* cultured and field grown roots of *W*. *somnifera* were analyzed using high performance thin layer chromatography. The accumulation pattern of withaferin A and withanolide A varied according to the type of tissue and the culture period. Significantly lower levels of withaferin A was observed in *in vitro* cultured roots than field grown roots (Table 1). Only leaf tissues preferentially accumulate withaferin A whereas *in vitro* adventitious root tissues accumulate prodigious quantities of withanolide A [31]. These results were coincide with the previous report that withaferin A accumulation was tightly linked only to leaf tissue rather root [32]. However, withanolide A contents in *in vitro* cultured roots were comparable to those of field grown roots. Significantly higher content of withanolide A (380.0±0.4 μg/g DW) was observed in 1.5MIR (*in vitro* roots cultured for 45 days) than 1MIR (*in vitro* roots cultured for 30 days). Among the field grown roots, higher content of withanolide A of 380.0±0.9 μg/g was obtained in 2MFR than 5MFR. Especially, withanolide A contents of 2MFR and 1.5MIR samples were similar to each other (380 μg /mL**)**. Considering the cultivation time, *in vitro* culture showed a better productivity than field grown samples. Hence, *in vitro* raised root cultures could serve as an alternative to field grown root for the production of withanolide A.

**Table 1:** Pattern of withanolide accumulation in *in vitro* and field grown tissues quantified using HPTLC.

**Sample** ^a^	**Withanolide A (μg/g dried weight)**	**Withaferin A (μg/g dried weight)**
2MFR	380.0±0.9^a^	180.0±1.2^a^
5MFR	287.0±0.9^b^	175.6±0.6^b^
1MIR	101.0±0.4^c^	2.4±0.8^c^
1.5MIR	380.0±0.4^a^	5.36±1.0^d^

The data presented in the figure are the mean ± standard deviation of three replicates obtained from three independent experiments. Different letters in the same column indicate a significant difference (*p*<0.05). Data represents Mean ± Standard deviation of three replicates obtained from three independent experiments.

^a^2MFR, 2 months field-grown root; 5MFR, 5 months field-grown root; 1MIR, 1MIR, 1 month *in vitro* root; 1.5 months *in vitro* root.

### 2. Free Radical Scavenging Activity, Total Phenolic and Flavonoid Content

It was previously reported that the methanol extracts of *W*. *somnifera* roots exhibited higher antioxidant activities compared to extracts from chloroform, acetone, and ethyl acetate [33]. Therefore, the free radical scavenging activity (FRSA) was investigated using methanol extracts of *W*. *somnifera* root samples. As listed in Table 2, the FRSA of the root samples revealed that 1MIR exhibited the highest activity with an IC_50_ value of 81.01 μg/mL, followed by 1.5MIR (IC_50_ 101.60 μg/mL), 5MFR (IC_50_ 188.40 μg/mL), and 2MFR (IC_50_ 117.27 μg/mL). Overall, the root samples cultivated *in vitro* showed significantly higher antioxidant potentials than the field samples. When we compare the root samples of 1MIR and 2MFR, it was revealed that 1MIR cultivated for a shorter period than 2MFR had a 1.3 times higher FRSA than that of 2MFR. Interestingly, the FRSA of *in vitro* cultured root samples decreased with longer cultivation periods (1MIR > 1.5MIR), while that of the field samples increased with longer cultivation periods (5MFR > 2MFR). The differences in FRSA according to cultivation period might be attributed to metabolic changes of active compounds in the samples. It has been reported that the FRSA of plant materials is strongly correlated with the amount of phenolics and flavonoids [34]. Therefore, the amount of phenolics and flavonoids in the samples was investigated.

**Table 2:** Free radical scavenging activity (FRSA), total phenolic contents (TPC) and total flavonoid content (TFC) of *W*. *somnifera* roots grown *in vitro* and in the field.

**Sample** ^a^	**FRSA IC**_50_ **(μg/mL)**	**TPC (mg GAE/g)**	**TFC (mg CE/g)**
2MFR	188.40 ± 3.83^a^	79.25 ± 0.84^a^	14.78 ± 0.33^a^
5MFR	117.27 ± 1.31^b^	95.45 ± 2.20^b^	19.84 ± 0.67^b^
1MIR	81.01 ± 1.60^c^	118.91 ± 2.85^c^	32.68 ± 0.46^c^
1.5MIR	101.60 ± 2.23^d^	104.40 ± 5.40^d^	22.03 ± 0.19^d^
Ascorbic acid	7.39 ± 0.08^e^	-	-

Each value is shown as the mean ± SD (N = 7). Different letters in the same column indicate a significant difference (*p*<0.05). GAE: gallic acid equivalent, CE: catechin equivalent.

^a^2MFR, 2 months field-grown root; 5MFR, 5 months field-grown root; 1MIR, 1MIR, 1 month *in vitro* root; 1.5 months *in vitro* root.

The total phenolic content (TPC) of the root samples was determined according to the Folin-Ciocalteu method and was expressed as gallic acid equivalents (GAE) per gram of extracted sample. The total flavonoid content (TFC) of the root sample was evaluated using a calibration curve with a catechin standard and was expressed as catechin equivalents (CE) per gram of extracted sample. The TPC and TFC of the root samples are summarized in Table 2. The TPC ranged from 79.3 to 118.9 GAE mg/g, and 1MIR (118.9 GAE mg/g) yielded the highest TPC followed by 1.5MIR (104.4 GAE mg/g), 5MFR (95.5 GAE mg/g), and 2MFR (79.3 GAE mg/g). Additionally, the TFC ranged from 14.8 to 32.7 CE mg/g, and 1MIR (32.7 CE mg/g) showed the highest TPC followed by 1.5MIR (22.0 CE mg/g), 5MFR (19.9 CE mg/g), and 2MFR (14.8 CE mg/g). Significantly higher TPC and TFC were observed in *in vitro* cultured roots, indicating that they could be used as an alternative to field root samples.

To elucidate the relationship between antioxidant activity, TPC, and TFC, Pearson’s correlation test was performed using the data set listed in S1 Table and the results are presented in Table 3. For correlation analysis, the IC_50_ values of FRSA were transformed into their reciprocal values (1/IC_50_). The FRSA of root samples were positively correlated with TPC (*r* = 0.974), and TFC (*r* = 0.953) with statistical significance of *p*<0.01 (Table 3). The significant correlations could be due to the use of methanol, which was reported to be an efficient solvent to extract phenolics and flavonoids from *W*. *somnifera* [35]. In addition, a significant correlation (*r* = 0.946, *p*<0.01) was found between TPC and TFC, indicating that flavonoids were the major components of phenolics in the root samples. It was reported that the gene cluster for phenylpropanoid biosynthesis accounted for the largest group in biosynthesis of secondary metabolites in *W*. *somnifera* roots [36]. It seems that the phenylpropanoid pathway might be more activated in *in-vitro* cultured *W*. *somnifera* roots. It was reported that 8-week-old callus induced from *W*. *somnifera* leaves dramatically accumulated proteins, soluble sugars, and lipids, but had less TPC than the leaves and roots of field plants [37]. In addition, it was reported that roots of *W*. *somnifera* cultured in solid medium showed higher levels of TPC and TFC than those acclimatized and grown in a greenhouse [38]. However, there had been no reports regarding investigation of FRSA and comparative metabolic profiling between *in vitro* cultured and field grown roots of *W*. *somnifera*. In this study, *in vitro* cultured roots showed a higher FRSA, TPC, and TFC than field grown roots.

**Table 3:** Pearson’s correlation coefficients (*p*<0.01) between antioxidant activity (FRSA), total phenolic content (TPC), and total flavonoid content (TFC) of the extracts from roots of *W*. *somnifera*.

	**FRSA**	**TPC**	**TFC**
FRSA	1	0.974	0.953
TPC		1	0.946
TFC			1

### 3. Identification of Metabolites and Principal Component Analysis (PCA)

As shown in Table 4, the following 29 metabolites were identified in *in vitro* cultured and field grown roots of *W*. *somnifera*: alcohols (glycerol, mannitol, myo-inositol, and xylitol), amino acids (β-alanine, asparagine, aspartic acid, glutamic acid, glutamine, glycine, lysine, phenylalanine, proline, serine, and threonine), organic acids (aconitic acid, citric acid, fumaric acid, glucaric acid, gluconic acid, glucuronic acid, glyceric acid, malic acid, succinic acid, and xylonic acid), phenolic acid (vanillic acid), sugars (fructose, and glucose), and putrescine.

**Table 4:** Metabolites identified in methanol extracts of *W*. *somnifera* roots by GC-MS.

Class	Metabolites	RT(min)	Fragmentation ion (m/z)	TMS	KEGG ID
Alcohols	Glycerol	10.23	73, 117, 147, 205, 308(M^+^)	3 TMS	C00116
	Mannitol	28.54	73, 147, 205, 319, 614(M^+^)	6 TMS	C00392
	Myo-inositol	33.36	73, 147, 217, 305, 612(M^+^)	6 TMS	C00137
	Xylitol	22.20	73, 147, 217, 307, 512(M^+^)	5 TMS	C00379
Amino acids	β-Alanine	14.16	73, 174, 248, 290, 305(M^+^)	3TMS	C00099
	Asparagine	20.66	73, 116, 231, 333, 348(M^+^)	3 TMS	C00152
	Aspartic acid	16.59	73, 100, 147, 218, 349(M^+^)	3 TMS	C00049
	Glutamic acid	19.30	73, 147, 246, 348, 363(M^+^)	3 TMS	C00025
	Glutamine	23.78	73, 156, 245, 347, 362(M^+^)	3 TMS	C00064
	Glycine	10.96	73, 147, 174, 276, 291(M^+^)	3 TMS	C00037
	Lysine	28.35	73, 174, 317, 419, 434(M^+^)	4 TMS	C00047
	Phenylalanine	37.99	73, 192, 218, 294, 309(M^+^)	2 TMS	C00079
	Proline	10.74	73, 142, 216, 244, 259(M^+^)	2 TMS	C00148
	Serine	12.47	73, 147, 204, 218, 321(M^+^)	3 TMS	C00065
	Threonine	13.12	73, 117, 218, 291, 335(M^+^)	3 TMS	C00188
Organic acids	Aconitic acid	23.17	73, 147, 229, 375, 390(M^+^)	3 TMS	C00417
	Citric acid	25.24	73, 147, 273, 465, 480(M^+^)	4 TMS	C00158
	Fumaric acid	12.25	73, 147, 217, 245, 260(M^+^)	2 TMS	C00122
	Glucaric acid	32.00	73, 147, 333, 627, 642(M^+^)	6 TMS	C00818
	Gluconic acid	30.67	73, 147, 205, 333, 628(M^+^)	6 TMS	C00257
	Glucuronic acid	42.08	73, 147, 204, 292, 554(M^+^)	5 TMS	C00191
	Glyceric acid	11.67	73, 147, 189, 292, 322(M^+^)	3 TMS	C00258
	Malic acid	15.81	73, 147, 233, 335, 350(M^+^)	3 TMS	C00149
	Succinic acid	11.27	73, 129, 147, 247, 262(M^+^)	2 TMS	C00042
	Xylonic acid	19.57	73, 147, 217, 349, 364(M^+^)	3 TMS	C05411
Phenolic acid	Vanillic acid	23.43	73, 223, 267, 297, 312(M^+^)	2 TMS	C06672
Sugars	Fructose	25.11	73, 103, 217, 307, 569(M^+^)	5 TMS	C00095
	Glucose	27.45, 28.87	73, 147, 204, 525, 540(M^+^)	6 TMS	C00031
Other	Putrescine	22.47	73, 174, 214, 361, 376(M^+^)	4 TMS	C00134

Each base peak in the mass spectra is identified by bold type. KEGG ID means that the metabolite could be identified in the KEGG pathway. M+, molecular ion peak; TMS, trimethylsilylation; *p*-value < 0.05.

**Fig 1:**
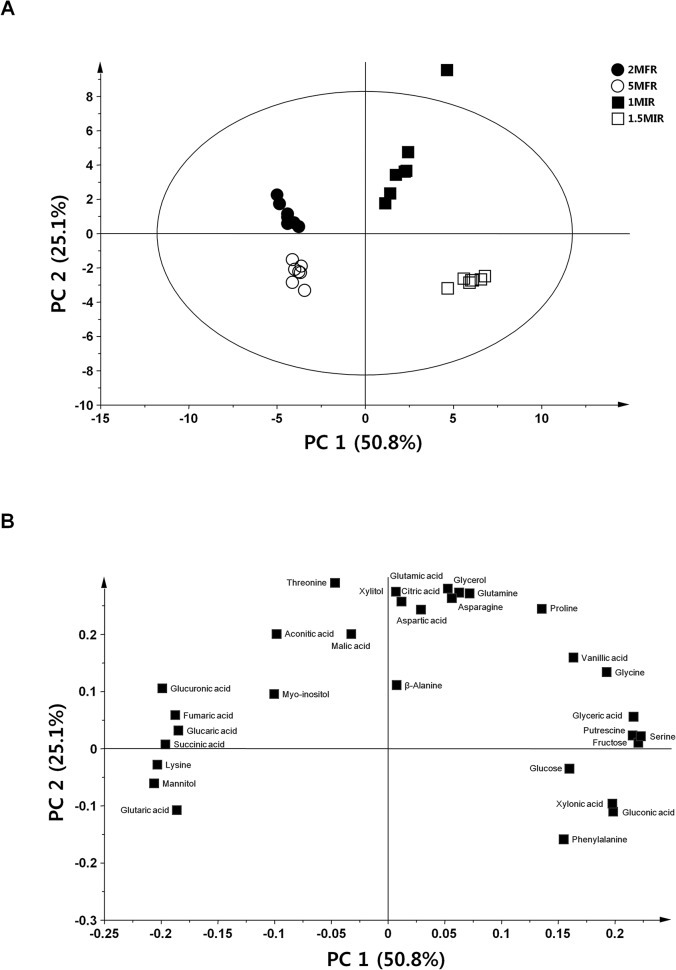
Principal component analysis (PCA) of the metabolomes derived from *in vitro* cultured and field grown roots of *W*. *somnifera*. (A) PCA-derived score plots. (B) PCA-derived loading plots. PC: principal component.

The PCA derived score plot derived from the analysis of *W*. *somnifera* roots is shown in Fig 1A. Each root sample was clearly separated by PC 1, accounting for 50.8% of the total variation, and by PC 2, accounting for 25.1% of the variation. Loading plots corresponding to the score plots was shown in Fig 1B. Most of amino acids were present at higher levels in the field root samples (2MFR and 5MFR) than in the *in vitro* root samples (1MIR and 1.5MIR). Among them, a phenylalanine as a precursor of polyphenol was contained much in *in vitro* root sample than in field root sample. Furthermore, it was revealed that significantly higher levels of vanillic acid, glucose, and fructose were existed in 1MIR and 1.5MIR root samples than in the field root samples.

### 4. Comparison of Metabolic Profiles of *in vitro* Cultured and Field Grown *W*. *somnifera* Roots

**Fig 2:**
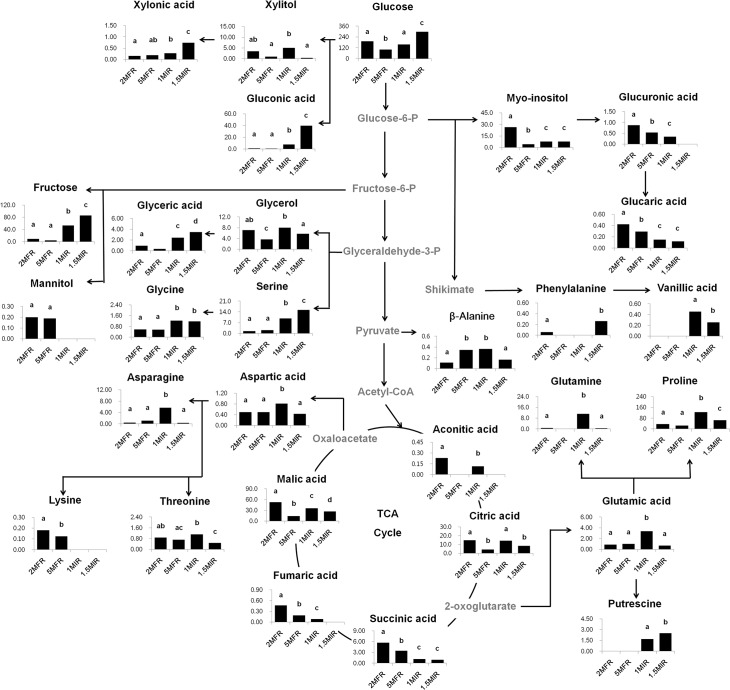
Schematic diagram of the metabolic pathway and relative levels of major compounds detected in *W*. *somnifera* root extracts. This was modified from the pathways presented in the KEGG database (http://www.genome.jp/kegg/). ANOVA was performed to assess the statistical significance of differences between samples (*p*-value < 0.05). Data are presented as mean values and error bars represent the standard deviation. Different letters represent the statistical significance of metabolite levels.

The relative levels of various metabolites were investigated, and biochemical changes throughout the metabolic pathway were shown in Fig 2 and S2 Table. ANOVA was performed to assess the statistical significance on relative level of each metabolite among different samples (*p*<0.05). Myo-inositol levels of 2MFR were significantly higher than those in *in vitro* grown root samples (1MIR and 1.5MIR). Accumulation of myo-inositol in the cytosol was observed during salt stress. Field-grown plants are adversely affected by salinity, a major environmental stress factor that limits agricultural production [39]. The increased level of myo-inositol in field grown 2MFR samples as compared to that of *in vitro* cultured samples might be attributed to a tolerance to decreased water levels or stress induced by high salinity levels. It has been also reported that myo-inositol could serve as a substrate for the production of d-ononitol and d-pinitol, which mediate tolerance to drought or high salt conditions [40]. Decreased myo-inositol levels of the root samples with longer cultivation periods could be due to the production of _D_-ononitol and _D_-pinitol from myo-inositol to adapt osmotic stress.

Proline was the most abundant amino acids in root samples. It has been reported that the accumulation of proline ameliorate osmotic stress [41]. In addition, proline has been known to protect membranes from damage induced by reactive oxygen species and has been associated with the prevention of protein denaturation, and the preservation of the structure and activity of essential enzymes [42–44]. Moreover, the functional significance of proline accumulation has been known to provide sufficient energy to sustain rapid cell growth [43]. It was found that amino acids can induce rhizogenesis. It was reported that proline especially increased the rooting percentage and number of roots per rooted explants of sweet cherry (*Prunus avium* L.) [45]. The significantly higher level of proline in 1MIR sample could be related to initial root growth of *in vitro* cultured *W*. *somnifera* root.

Vanillic acid was only detected in *in vitro* cultured roots (1MIR and 1.5MIR) and the levels of vanillic acid were higher in 1MIR than 1.5MIR. The biosynthesis of phenolic metabolites such as vanillic acid has been known to be stimulated by activation of phenylpropanoid biosynthetic pathway through proline linked pentose phosphate pathway. It has been proposed that the proline biosynthesis was closely associated with the pentose phosphate pathway [44]. The accumulation of vanillic acid in *in vitro* cultured root samples might be stimulated by the pentose phosphate pathway linked to increased proline levels. In addition, it is well known that vanillic acid has a significant antioxidative activity from *in vitro* experiments [46,47]. The highest level of vanillic acid in *in vitro* sample 1MIR than other samples could contribute to the most active antioxidant activity of 1MIR sample.

The levels of soluble sugars, including fructose and glucose, were significantly different between the field grown and *in vitro* cultured samples. The sugar levels of *in vitro* cultured root samples were much higher than those of field grown root samples. Li et al (2013) [48] observed similar result of high sugar levels released by peanut root cultures and concluded that accumulation of sugar molecules could provide abundant nutrition for growth and development.

## Conclusion

Withanolides, signature metabolite of *W*. *somnifera* appear to be present in varying concentration at different period of growth. Further, enhancement of withanolide accumulation can be done upon exposure of *in vitro* root cultures to elicitors and abiotic stress conditions, since, field grown materials are highly affected by genotype and adverse environmental conditions. The present study compared the free radical scavenging activity (FRSA), total phenolic (TPC), total flavonoid contents (TFC), and metabolic profiles of field-grown and *in vitro* cultured root samples of *W*. *somnifera*. I*n vitro* cultured roots showed a higher FRSA, TPC, and TFC than field grown roots. Specifically, roots cultivated for 30 days (1MIR) showed higher FRSA than those cultivated for 45 days (1.5MIR). As the correlations between the FRSA, TPC, TFC were found to be statistically significant (*p*<0.01), phenolic compounds including flavonoids appear to be responsible for the highest antioxidant activity of 1MIR. The results of this study highlighted the potential utilization of *in vitro*-cultured *W*. *somnifera* roots as alternative resources to field-grown roots, as they exhibited relatively higher antioxidant activities and shorter cultivation periods. Moreover, *in vitro*-cultured *W*. *somnifera* roots had advantages for the production of useful metabolites such as withanolide A and vanillic acid. This study implies that *in vitro* cultured roots of *W*. *somnifera* can be used for the development of biopharmaceuticals or functional foods.

## Supporting Information

S1 Table Free radical scavenging activity (FRSA), total phenolic contents (TPC) and total flavonoid content (TFC) of *W*. *somnifera* roots grown *in vitro* and in the field. Seven independent sample extracts of *W*. *somnifera* roots cultivated under different environment were prepared and determined.
(DOCX)

S2 Table GC-MS-based metabolic profiling of 70% methanol extracts of *W*. *somnifera* roots. The relative levels of each metabolite were obtained by dividing the percentage area corresponding to each metabolite by the percentage area of the internal standard. Different letters in the same row indicate a significant difference. Mean ± SD values (n = 7) are shown. “ND” means “not detected.”
(DOCX)
