# Supplementary material for: Free Radical Scavenging Activity and Comparative Metabolic Profiling of In Vitro Cultured and Field Grown Withania somnifera Roots
Source: PLoS One. 2015 Apr 14;10(4):e0123360. doi: 10.1371/journal.pone.0123360 (PMC4397045; doi:10.1371/journal.pone.0123360)
Supplement: S1 Table — Seven independent sample extracts of W. somnifera roots cultivated under different environment were prepared and determined. (DOCX) [file pone.0123360.s001.docx]

**S1 Table. Free radical scavenging activity (FRSA), total phenolic contents (TPC) and total flavonoid content (TFC) of *W. somnifera* roots grown *in vitro* and in the field.** Seven independent sample extracts of *W. somnifera* roots cultivated under different environment were prepared and determined.

| \| **Sample**^a^ \| **FRSA**  **IC_50_ (µg/mL)** \| **TPC**  **(mg GAE/g)**^b^ \| **TFC**  **(mg CE/g)**^c^ \| \| --- \| --- \| --- \| --- \| \| 2MFR \| 194.71 \| 77.82 \| 14.52 \| \| 187.74 \| 79.70 \| 14.52 \| \| 186.92 \| 79.25 \| 15.14 \| \| 190.26 \| 79.70 \| 14.52 \| \| 185.34 \| 79.70 \| 15.14 \| \| 190.66 \| 78.41 \| 14.52 \| \| 183.14 \| 80.18 \| 15.14 \| \| 5MFR \| 117.40 \| 97.44 \| 18.91 \| \| 119.66 \| 97.44 \| 19.50 \| \| 117.31 \| 92.81 \| 19.50 \| \| 117.81 \| 96.32 \| 20.68 \| \| 116.54 \| 92.81 \| 19.50 \| \| 115.41 \| 93.89 \| 20.08 \| \| 116.73 \| 97.44 \| 20.68 \| \| 1MIR \| 81.12 \| 116.57 \| 32.19 \| \| 84.42 \| 118.82 \| 33.05 \| \| 80.96 \| 117.26 \| 33.05 \| \| 79.70 \| 122.18 \| 33.05 \| \| 80.27 \| 114.84 \| 32.19 \| \| 79.89 \| 120.52 \| 32.19 \| \| 80.68 \| 122.18 \| 33.05 \| \| 1.5MIR \| 99.36 \| 99.08 \| 21.82 \| \| 98.32 \| 101.08 \| 22.18 \| \| 102.33 \| 108.01 \| 21.82 \| \| 102.69 \| 99.22 \| 21.82 \| \| 100.61 \| 111.14 \| 22.18 \| \| 103.53 \| 110.85 \| 22.18 \| \| 104.39 \| 101.44 \| 22.18 \| |  |  |  |
| --- | --- | --- | --- | --- | --- | --- | --- | --- | --- | --- | --- | --- | --- | --- | --- | --- | --- | --- | --- | --- | --- | --- | --- | --- | --- | --- | --- | --- | --- | --- | --- | --- | --- | --- | --- | --- | --- | --- | --- | --- | --- | --- | --- | --- | --- | --- | --- | --- | --- | --- | --- | --- | --- | --- | --- | --- | --- | --- | --- | --- | --- | --- | --- | --- | --- | --- | --- | --- | --- | --- | --- | --- | --- | --- | --- | --- | --- | --- | --- | --- | --- | --- | --- | --- | --- | --- | --- | --- | --- | --- | --- | --- | --- | --- | --- |
| ^a^2MFR, 2 months field-grown root; 5MFR, 5 months field-grown root; 1MIR, 1MIR, 1 month *in vitro* root; 1.5 months *in vitro* root.  ^b^GAE: gallic acid equivalent  ^c^CE: catechin equivalent. |  |  |  |
