# Supplementary material for: Free Radical Scavenging Activity and Comparative Metabolic Profiling of In Vitro Cultured and Field Grown Withania somnifera Roots
Source: PLoS One. 2015 Apr 14;10(4):e0123360. doi: 10.1371/journal.pone.0123360 (PMC4397045; doi:10.1371/journal.pone.0123360)
Supplement: S2 Table — The relative levels of each metabolite were obtained by dividing the percentage area corresponding to each metabolite by the percentage area of the internal standard. Different letters in the same row indicate a significant difference. Mean ± SD values (n = 7) are shown. “ND” means “not detected.” (DOCX) [file pone.0123360.s002.docx]

**S2 Table. GC-MS-based metabolic profiling of 70% methanol extracts of *W. somnifera* roots.** The relative levels of each metabolite were obtained by dividing the percentage area corresponding to each metabolite by the percentage area of the internal standard. Different letters in the same row indicate a significant difference. Mean ± SD values (n = 7) are shown. “ND” means “not detected.”

| **Compound** | **RT**  **(min)** | **Relative intensity** | | | |
| --- | --- | --- | --- | --- | --- |
|  |  | **2MFR** | **5MFR** | **1MIR** | **1.5MIR** |
| **Alcohols** |  |  |  |  |  |
| Glycerol | 10.23 | 7.06 ± 0.24^ab^ | 3.65 ± 0.30^c^ | 8.00 ± 0.96^b^ | 5.75 ± 0.27^a^ |
| Mannitol | 28.54 | 0.20 ± 0.02^a^ | 0.19 ± 0.02^a^ | ND | ND |
| Myo-inositol | 33.36  33.55  42.66 | 26.42 ± 1.48^a^ | 4.43 ± 0.37^b^ | 7.90 ± 1.14^c^ | 7.69 ± 0.46^c^ |
| Xylitol | 22.20  27.38 | 3.47 ± 0.15^ab^ | 0.93 ± 0.12^a^ | 6.45 ± 3.18^b^ | 0.33 ± 0.05^a^ |
| **Amino acids** |  |  |  |  |  |
| Alanine | 14.16 | 0.11 ± 0.01^a^ | 0.35 ± 0.03^b^ | 0.36 ± 0.04^b^ | 0.17 ± 0.01^a^ |
| Asparagine | 20.66 | 0.40 ± 0.08^a^ | 1.11 ± 0.19^a^ | 5.68 ± 0.70^b^ | 0.34 ± 0.03^a^ |
| Aspartic acid | 16.59 | 0.50 ± 0.07^a^ | 0.49 ± 0.10^a^ | 0.81 ± 0.17^b^ | 0.43 ± 0.02^a^ |
| Glutamic acid | 19.30 | 0.87 ± 0.28^a^ | 1.00 ± 0.24^a^ | 3.36 ± 0.90^b^ | 0.68 ± 0.06^a^ |
| Glutamine | 23.78 | 0.69 ± 0.17^a^ | ND | 11.25 ± 7.96^b^ | 0.58 ± 0.08^a^ |
| Glycine | 10.96 | 0.57 ± 0.06^a^ | 0.55 ± 0.07^a^ | 1.24 ± 0.16^b^ | 1.17 ± 0.09^b^ |
| Lysine | 28.35 | 0.18 ± 0.02^a^ | 0.12 ± 0.01^b^ | ND | ND |
| Phenylalanine | 37.99 | 0.06 ± 0.01^a^ | ND | ND | 0.26 ± 0.02^b^ |
| Proline | 10.74  16.46 | 35.75 ± 3.20^a^ | 25.80 ± 5.44^a^ | 125.28 ± 20.27^b^ | 64.62 ± 2.63^c^ |
| Serine | 12.47 | 1.40 ± 0.14^a^ | 2.11 ± 0.37^a^ | 9.63 ± 2.85^b^ | 15.20 ± 0.61^c^ |
| Threonine | 13.12 | 0.87 ± 0.10^ab^ | 0.71 ± 0.10^ac^ | 1.11 ± 0.15^b^ | 0.47 ± 0.02^c^ |
| **Organic acids** |  |  |  |  |  |
| Aconitic acid | 23.17 | 0.23 ± 0.02^a^ | ND | 0.12 ± 0.02^b^ | ND |
| Citric acid | 25.24 | 14.74 ± 1.53^a^ | 4.37 ± 0.55^b^ | 14.31 ± 3.62^a^ | 8.40 ± 0.50^b^ |
| Fumaric acid | 12.25 | 0.46 ± 0.03^a^ | 0.19 ± 0.02^b^ | 0.08 ± 0.01^c^ | ND |
| Glucaric acid | 32.00 | 0.42 ± 0.04^a^ | 0.29 ± 0.05^b^ | 0.15 ± 0.04^c^ | 0.12 ± 0.01^c^ |
| Gluconic acid | 30.67 | 1.03 ± 0.09^a^ | 0.42 ± 0.09^a^ | 7.57 ± 2.66^b^ | 39.73 ± 2.50^c^ |
| Glucuronic acid | 42.08 | 0.88 ± 0.08^a^ | 0.53 ± 0.05^b^ | 0.34 ± 0.06^c^ | ND |
| Glyceric acid | 11.67 | 0.95 ± 0.04^a^ | 0.34 ± 0.03^b^ | 2.43 ± 0.28^c^ | 3.48 ± 0.17^d^ |
| Malic acid | 15.81 | 52.29 ± 2.43^a^ | 13.99 ± 1.59^b^ | 35.48 ± 5.31^c^ | 26.61 ± 1.33^d^ |
| Ribonic acid | 24.04 | 0.36 ± 0.02^a^ | ND | 0.46 ± 0.10^a^ | 0.49 ± 0.03^a^ |
| Rythronic acid | 17.19 | 0.71 ± 0.03^a^ | 0.30 ± 0.02^a^ | 2.60 ± 0.33^b^ | 6.26 ± 0.35^c^ |
| Succinic acid | 11.27 | 5.68 ± 0.36^a^ | 3.44 ± 0.29^b^ | 1.14 ± 0.13^c^ | 0.92 ± 0.04^c^ |
| Threonic acid | 14.08 | ND | ND | 0.28 ± 0.04^a^ | 1.10 ± 0.06^b^ |
| Xylonic acid | 19.57 | 0.16 ± 0.01^a^ | 0.18 ± 0.03^ab^ | 0.28 ± 0.07^b^ | 0.74 ± 0.04^c^ |
| **Phenolic acid** |  |  |  |  |  |
| Vanillic acid | 23.43 | ND | ND | 0.45 ± 0.08^a^ | 0.25 ± 0.03^b^ |
| **Sugars** |  |  |  |  |  |
| Fructose | 25.11  26.04  26.38  26.93  27.22 | 8.75 ± 0.94^a^ | 4.08 ± 0.70^a^ | 53.08 ± 9.37^b^ | 86.17 ± 4.77^c^ |
| Galactose | 25.73  32.73  38.30  40.30  42.24  45.13  51.10  53.02  57.64 | 1.26 ± 0.07^a^ | 0.50 ± 0.06^b^ | 1.22 ± 0.26^a^ | 3.01 ± 0.13^c^ |
| Glucosamine | 28.22 | ND | ND | 3.33 ± 0.81^a^ | 9.34 ± 0.47^b^ |
| Glucose | 27.45  28.87  30.37  48.90  54.88  56.15  56.26  58.33 | 192.42 ± 5.57^a^ | 100.26 ± 8.83^b^ | 156.84 ± 27.39^a^ | 296.88 ± 15.42^c^ |
| Mannose | 39.53 | 0.05 ± 0.01^a^ | 0.14 ± 0.03^b^ | ND | ND |
| Melibiose | 53.96  54.37  55.42  57.45  58.19 | 0.58 ± 0.05^a^ | 1.31 ± 0.14^a^ | 4.36 ± 0.62^b^ | 4.98 ± 0.23^b^ |
| Ribose | 19.98 | 0.06 ± 0.00^a^ | 0.11 ± 0.02^b^ | 0.06 ± 0.01^a^ | 0.17 ± 0.01^c^ |
| Xylose | 45.72  50.96  53.49  54.70  55.02  56.83  57.35 | 2.81 ± 0.14^a^ | 1.90 ± 0.20^b^ | 2.15 ± 0.37^b^ | 1.29 ± 0.07^c^ |
| **Other** |  |  |  |  |  |
| Putrescine | 22.47 | ND | ND | 1.69 ± 0.22^a^ | 2.49 ± 0.16^b^ |
